# Nanoparticles of non-porphyrinic covalent organic frameworks as contrast agents for photoacoustic imaging

**DOI:** 10.1038/s41598-025-08631-w

**Published:** 2025-09-30

**Authors:** Irene Pi-Martín, Carla Vidaurre-Agut, Eva María Rivero-Buceta, Alejandro Cebrecos, Juan José García-Garrigós, Noé Jiménez, José María Benlloch, Pablo Botella, Francisco Camarena

**Affiliations:** 1 Instituto de Instrumentación para Imagen Molecular (i3M), Universitat Politècnica de València - Consejo Superior de Investigaciones Científicas, Avenida de los Naranjos s/n, 46022 Valencia, Spain; 2 Instituto de Tecnología Química, Universitat Politècnica de València - Consejo Superior de Investigaciones Científicas, Avenida de los Naranjos s/n, 46022 Valencia, Spain

**Keywords:** Photoacoustic imaging, Exogenous contrast agents, Covalent organic frameworks, Nanoparticles, Characterization and analytical techniques, Imaging techniques

## Abstract

Photoacoustic imaging (PAI) is an emerging biomedical modality offering non-invasive, high-resolution imaging with molecular contrast. Its effectiveness for molecular imaging is improved by using external contrast agents such as dyes and nanostructures, which enable targeted imaging, deeper tissue penetration, and enhanced diagnostic accuracy. However, the clinical translation of these agents has been hindered by limitations in biocompatibility, biodegradability, and functionalization versatility. In this study, two porphyrin-free nanoscale covalent organic frameworks (nCOFs), TAPB-PDA and LZU-1, were evaluated as novel exogenous contrast agents for PAI. Their photoacoustic responses were systematically measured and compared with traditional contrast agents, such as gold nanoparticles (AuNPs) and ATTO532-labelled mesoporous silica nanoparticles. Experiments at 532 nm across a range of concentrations demonstrated strong photoacoustic signals from TAPB-PDA and LZU-1, with enhanced response at higher concentrations. Besides, both nCOFs also provided detectable signals at the NIR window at the lowest concentration, indicating a broad working range. A nonlinear model of the volume-integrated photoacoustic pressure is also provided. Furthermore, in vitro cytotoxicity assays indicated excellent nCOF biocompatibility, even at higher concentrations. Photostability tests under prolonged pulsed laser irradiation revealed that both nCOFs exhibit good resistance to photobleaching and partial signal recovery under intermittent excitation. Collectively, these findings highlight the potential of TAPB-PDA and LZU-1 nCOFs as biocompatible, biodegradable, and functionally versatile contrast agents for advanced photoacoustic imaging.

## Introduction

Photoacoustic imaging (PAI) is an advanced imaging modality with significant applications in the biomedical field, as it enables the acquisition of both functional and molecular information about biological systems, serving as a tool for both diagnosis and treatment monitoring^1,2^. Image contrast in PAI is derived from the optical absorption properties of endogenous chromophores, such as haemoglobin, lipids, and melanin, among others, when stimulated by nanosecond-pulsed laser light. While these endogenous chromophores provide intrinsic contrast, the use of exogenous contrast agents is gaining relevance in PAI. These externally introduced chromophores can significantly improve image quality (contrast and penetration depth), and offer enhanced sensitivity and specificity, enabling targeted imaging of molecular and cellular processes^3–5^. As an engineered chromophore, an excellent contrast agent should possess the photophysical properties of low quantum yield, high molar extinction coefficient, peak absorption in the NIR window and excellent photostability, as well as low toxicity and immunogenicity, high target affinity and specificity, and high biocompatibility^6^.

Exogenous agents utilized in photoacoustic imaging can be broadly categorized into two main groups^3^: small-molecule dyes^7–11^ and nanostructures, which are further classified as either metallic^12–22^ or organic^23–28^. The incorporation of nanoparticles significantly expands the scope of this imaging modality^29^, as they are often engineered to exhibit peak optical absorption in the near-infrared region (NIR), where tissue optical attenuation is typically low, thereby enabling deeper penetration of light into the tissue^30^. Dye-loaded nanoparticles leverage a high concentration of NIR organic dyes to enhance optical absorption^31^. While these dyes exhibit low toxicity, favourable biodistribution, efficient clearance, and, in some cases, clinical approval, their limited photostability and susceptibility to degradation in aqueous environments significantly restrict their biomedical applications. In the context of nanostructures, some possess relatively simple architectures, such as metallic nanospheres, which accumulate in tumours through passive targeting mechanisms^32^. Conversely, more complex nanostructures are specifically engineered to actively target specific biomolecules or function as carriers for therapeutic agents, further broadening their potential applications in biomedical imaging and treatment^33,34^.

Gold and silica nanoparticles have been extensively investigated and even evaluated in human subjects for a range of biomedical applications, including photothermal therapy and targeted drug delivery^35,36^. Gold nanoparticles, in particular, have demonstrated potential in clinical trials for localized tumour ablation owing to their strong photoacoustic response and biocompatibility at low doses^6,37^. Similarly, mesoporous silica nanoparticles have been utilized both as imaging agents and as carriers for controlled drug release^38^, benefiting from tunable surface chemistry and stability in biological environments^39^. Despite these advantages, inorganic nanoparticles exhibit inherent limitations, including the risk of long-term accumulation, induction of oxidative stress^40–43^ and potential toxicity at higher doses, which constrain their suitability for repeated or high-dose applications^44,45^.

Consequently, the development of new contrast agents able to promote PAI with high stability in biological fluids, good biocompatibility and a suitable biodegradation profile is needed. In this context, covalent organic frameworks (COFs) are a novel type of materials with topologically ordered columnar $\pi$ alignment^46^. COFs present unique structural periodicity that allows modulation of photoacoustic properties, as well as outstanding tunability of pore dimensions and functionalization. In particular, imine-based COFs with a laminar structure have demonstrated strong photostability when functioning as photocatalysts in oxidative transformations, exhibiting high performance and negligible degradation during consecutive catalytic runs^47^.

In addition, their nature is fully organic (body related), some compositions present strong stability in physiological fluids, they are non-cytotoxic and biodegradable over a wide range of concentrations. Very recently, it has been pointed out that these materials present photoacoustic properties highly dependent on their layered structures^27,48–51^. However, most of these examples are based on porphyrin-based COFs^27,52–56^, which are suspected to present significant in vivo toxicity^57^.

In this article, we assess the feasibility of porphyrin-free covalent organic framework nanoparticles (nCOFs) as exogenous contrast agents for PAI. Specifically, nanoparticles of the LZU-1 and TAPB-PDA materials^58,59^, with 2D-$\pi$ conjugation, have been evaluated for photoacoustic tomography with a hybrid PA and ultrasound (US) imaging system. Their photoacoustic (PA) response has been compared with standard nanoparticles, such as mesoporous silica nanoparticles labelled with the chromophore ATTO532 (MSN-ATTO532) and gold nanoparticles (AuNPs) at 532 nm for increasing concentrations and along the NIR spectral range at low concentration. Photostability of all contrast agents was also assessed under repeated pulsed laser excitation, revealing that LZU-1 and TAPB-PDA exhibit good stability as well as partial signal recovery under intermittent illumination. Finally, the cytotoxicity of both LZU-1 and TAPB-PDA nanoparticles has been evaluated in NIH/3T3 fibroblasts and HeLa human cancer cells, demonstrating high cell viability (>85%) across a range of concentrations. These findings confirm their biocompatibility and support their potential for safe use in biomedical applications, including molecular imaging and theranostics. The obtained results show that both nCOFs show promising behaviour as contrast agents for PAI, which is dependent on the interlayer stacking of their 2D-structure.

## Methods

### Materials

All reagents were purchased from commercial suppliers and used without further purification. 1,3,5-Tris(4-aminophenyl) benzene (TAPB), 1,3,5-triformylbenzene (TFB) were purchased from TCI. Terephthaldehyde (PDA), 3-aminopropyltrimethoxysilane (APTMS), ATTO532 and scandium triflate were obtained from Sigma Aldrich. 4-(tert-butoxycarbonylamino)-aniline (NBPDA) was purchased from Fluorochem. Poly(vinylpyrrolidone) (PVP) was acquired from Fluka. Anhydrous acetonitrile (ACN), anhydrous dichloromethane (DCM) and anhydrous toluene were obtained from a solvent purification system (MB SPS-800). AuNPs of 20 nm mean diameter, stabilized in citrate buffer were supplied by Sigma-Aldrich.

### Synthesis of nanoparticles

For the preparation of TAPB-PDA nanoparticles, we followed an optimized recipe from Ref.^25^. Briefly, 149 mg (0.424 mmol) of TAPB and 85 mg (0.634 mmol) of PDA were added to anhydrous acetonitrile (100 mL) and the solution was sonicated 2 min to dissolve the monomers. Scandium triflate (100 mg, 0.16 equiv) was subsequently added and sonicated for 20 s to dissolve and disperse the catalyst. The reaction mixture was left at room temperature and reacted for 72 h. Afterwards, the solid was precipitated with 5 mL of brine solution and filtered off. Later, nanoparticles were washed in a Soxhlet with 100 mL of methanol. The solid was dried overnight at room temperature under vacuum.

For the synthesis of LZU-1 nanoparticles, a modified protocol was implemented^59^. Here, 150 mg (0.925 mmol) of TFB, 300 mg (1.441 mmol) of NBPDA and 600 mg PVP (Mw = 360,000) were dissolved in 18 mL ethanol and 2 mL of trifluoroacetic acid (TFA). The solution was inserted in a 50 mL glass microwave vessel and heated to 120 $^\circ$C for 60 minutes (Biotage Initiator+). The reddish dispersion obtained was diluted with 13 mL of ethanol and centrifuged at 15,000 rpm for 15 minutes. The red solid collected was dispersed in 25 mL ethanol and 2 mL of triethylamine (TEA) to give a yellow suspension. The solution was centrifuged again at 15,000 rpm to collect the nanocrystals and the supernatant was decanted. The solid was dried overnight at room temperature under vacuum.

The preparation of ATTO532 labelled nanoparticles was carried out by post-synthetic modification of mesoporous silica nanoparticles (MSN)^60^. Firstly, amino-derivatized MSN (MSN-NH2) were prepared by surface functionalization with 3-aminopropyl-trimethoxysilane (APTMS). MSN (500 mg) was dried at 350 $^\circ$C and vacuumed for 3 h. Subsequently, 20 mL of anhydrous toluene was added, and the mixture was heated to reflux. Then, 975 $\upmu$L of APTMS (4.2 mmol) was introduced and the mixture was stirred for 3 h. The obtained product was filtered off, washed with toluene and methanol, and freeze-dried (− 55 $^\circ$C, 16 h). Later, 50 mg of MSN-NH2 were suspended in 10 mL of anhydrous dichloromethane (DCM) and 500 $\upmu$L of ATTO532 solution in DMSO (1.25 mg/ml) was injected under nitrogen atmosphere. The reaction was stirred for 3 h at room temperature without light. Afterwards, the nanoparticles were filtered off and washed with DCM. Finally, the sample was freeze-dried.

### Characterization of nanoparticles

Powder X-ray diffraction (XRD) patterns were collected on a Philips X’Pert diffractometer equipped with a graphite monochromator, operating at 40 kV and 45 mA. Nanoparticle morphology was investigated by transmission electron microscopy (TEM) on a JEOL JEM-1400 Flash microscope operating at 120 kV. The size distribution of the synthesized nanoparticles in aqueous dispersions was determined using a Zetasizer Nano ZS (Malvern Instruments Ltd., Worcestershire, United Kingdom). Here, solutions of dried materials in ACN (nCOFs) or deionized water (MSN-ATTO532) at a concentration of 5 $\upmu$g/mL were prepared and dynamic light scattering (DLS) measurements were performed at 25 $^\circ$C and $173^\circ$ scattering angle. Liquid nitrogen adsorption-desorption isotherms were measured in a Micromeritics ASAP 2420 System apparatus. The pore size distribution was calculated according to the Barret-Joyner-Halenda (BJH) algorithm. Surface area calculations were carried out using the Brunauer-Emmett-Teller (BET) method. Diffuse reflectance UV–Vis spectra (DRS) were collected on an Agilent Cary 7000 spectrophotometer equipped with a ‘Praying Mantis’ attachment (from Harrick) under ambient conditions. Thermal stability of the as-synthesized materials was monitored by thermogravimetric analysis (TGA) on a Mettler-Toledo TGA/SDTA 851e apparatus. The formation of the imine network was monitored using Fourier transform infrared (FTIR) spectroscopy. FTIR spectra were recorded at room temperature in the 400–4000 cm^-1^ region with a Nicolet 205xB spectrophotometer, equipped with a Data Station, at a spectral resolution of 1 cm^-1^ and accumulations of 128 scans.

### Photoacoustic system

**Figure 1:**
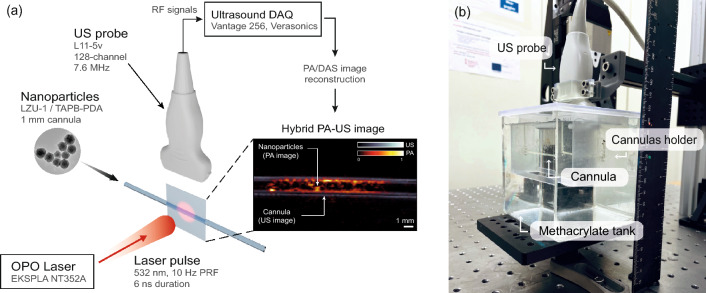
Hybrid PA-US system. (**a**) Schematic diagram of the experimental setup. Inset illustrates an experimental PA-US image of a cannula containing nanoparticles. (**b**) Image of the experimental setup.

The PAI system consists of an Optical Parametric Oscillator (OPO) tunable laser (EKSPLA NT352A, Vilnius, Lithuania), emitting pulses at 10 Hz repetition rate with 6 ns duration, which is synchronized with a Verasonics Vantage 256^TM^ acquisition system connected to an ultrasound linear probe (L11-5v, Verasonics) of 5–11 MHz bandwidth and 128 elements. Figure 1a shows a schematic diagram of the experimental setup used to obtain the photoacoustic (PA) response of the nanoparticles. The samples under study were diluted in phosphate-buffered saline (PBS, pH 7.4) to different concentrations, placed inside plastic cannulas (1.0 mm inner/2.0 mm outer diameter) parallel to the ultrasound (US) probe and held in a custom designed plastic holder, centered on the laser beam and immersed to a depth of 25 mm in deionized water, as shown in Fig. 1b. The collimated laser beam has a Gaussian cross-section of 5 mm diameter (at 1/$\hbox {e}^2$) which was enlarged three times, up to 15 mm, by an achromatic beam expander (GBE03, Thorlabs) and delivered to the cannula containing the nanoparticle samples at a constant laser pulse energy of 60 mJ, yielding an average optical fluence incident to the samples of around 34 mJ/cm^2^. In this regard, experiments were carried out using an initial set of 40 laser pulses to stabilize the emitted energy. Then, PA and US signals were averaged over 20 additional laser excitations.

The acquisition system co-registers data to generate hybrid PA-US images using the same US probe, as shown in the inset of Fig. 1a, ensuring spatial alignment in the co-registration. Delay-and-Sum (DAS) reconstruction was performed for both types of images using the same speed of sound, to maximize temporal alignment of the hybrid system. The US imaging provides the location and geometry of the cannula within the imaging space and the PAI provides the PA response of the illuminated nanoparticles. The PA signal amplitude was calculated as the spatial average of the reconstructed photoacoustic pressure inside the cannula. The co-registered US image was used to define a mask to select the image area corresponding to the nanoparticle solution, including a safety margin within the cannula walls.

### Photoacoustic response at 532 nm

Initially, we assess whether the amplitude of the PA response of the proposed organic nanoparticles is comparable to that of a reference standard, namely commercially available gold nanoparticles (AuNPs). The PA response is evaluated in comparison to LZU-1 and TAPB-PDA nCOFs, as well as mesoporous silica nanoparticles labelled with the ATTO532 fluorochrome (MSN-ATTO532), under laser excitation at 532 nm, which corresponds to the peak absorption wavelength of AuNPs and MSN-ATTO532. To ensure consistency, all samples were diluted to a concentration of 0.1 mg/mL, which represents the maximum available concentration for the commercial AuNPs. The baseline PA signal was obtained from measurements conducted on a control sample consisting of a cannula filled with PBS in the absence of nanoparticles. Subsequently, the PA response was evaluated for increasing concentrations (0.1, 0.5, 1, 2, 3, 4, and 5 mg/mL) of LZU-1, TAPB-PDA, and MSN-ATTO532 nanoparticles under excitation at a laser wavelength of 532 nm. It is important to note that the maximum concentration available for the AuNPs was 0.1 mg/mL, hence these samples were not included in this analysis. Additionally, the measured PA signal amplitudes at varying concentrations were fitted to a theoretical model describing the underlying PA effect.

### Photostability

Photostability of all four contrast agents, namely: AuNPs, LZU-1, TAPB-PDA and MSN-ATTO532, was evaluated at a concentration of 0.1 mg/mL using the experimental setup shown in Fig. 1. The excitation protocol consisted of 15 consecutive sets of 2,020 laser pulses (PRF = 10 Hz) at 532 nm, with an average optical fluence of 34 mJ/cm^2^, for a total of 30,300 laser pulses. After each set of laser pulses, the laser was stopped and remained in idle state for 60 s. Note that the first 40 laser pulses of each set were removed from the analysis to guarantee a stable emitted energy. The PA signal amplitudes were normalized to the maximum value of each sample to allow for direct comparison across agents. Finally, the decay curves obtained for the first set of laser pulses were fitted to a double-exponential model of the form $A \exp (-k_1x) + B \exp (-k_2 x)$, from which a global photothermal bleaching rate $k = A k_1 + B k_2$ was extracted for each case^61^.

### NIR photoacoustic spectroscopy

A near-infrared (NIR) photoacoustic spectroscopy analysis was conducted to assess whether these compounds exhibit a sufficient PA response for potential application within the NIR optical window. This spectral region is characterized by reduced light scattering compared to the visible range, thereby enabling greater tissue penetration. For this evaluation, all samples were prepared at a concentration of 0.1 mg/mL, and a laser excitation scan was performed over a wavelength range of 660 nm to 940 nm in increments of 20 nm.

### Theoretical model of the photoacoustic response

In this section, an integrated nonlinear model of the photoacoustic response depending on the light attenuation (absorption and scattering) across a sample volume is derived to provide useful theoretical expressions for general PA experimental measurements. The linear regime approximation of the full model was used as a fitting framework for characterizing the PA response across varying concentrations of nanoparticles.

#### Photoacoustic pressure depending on optical fluence attenuation

Photoacoustic measurements are generally performed by recording the signal amplitude from an ultrasonic probe. This amplitude is directly proportional to the laser-induced pressure, generated by rapid thermoelastic expansion that results from the absorption of short optical pulses. Thus, the initial pressure distribution, $p_0 (r)$, at each spatial point, $r = (x, y, z)$, is described by the following time-independent photoacoustic relation^1,62^

1 $$\begin{aligned} p_0 (r) = \Gamma H(r) = \Gamma \eta _{th} E_a(r) = \Gamma \eta _{th} \mu _a (r) \varphi _i (x,y) \exp (-\mu^{\prime}_t z). \end{aligned}$$

This expression (in the first equality) essentially states that the initial pressure is proportional to the heat, *H*(*r*) (energy density units, [J/$\hbox {m}^3$]). The heat is produced by optical absorption and is converted into pressure according to the dimensionless Grüneisen coefficient, $\Gamma$, depending on the thermodynamic properties of the medium. In the next equality, $\eta _{th}$, represents the photothermal conversion efficiency of the absorbed optical energy, $E_a$, accounting for nonthermal relaxation processes of the medium molecules such as the fluorescence radiative process. This time-independent relation holds for laser pulse widths shorter than the thermal and stress confinement characteristic times (typically around 100 ns).

The right-hand side of Eq. (1) then expresses the absorbed energy as the product of the absorption coefficient, $\mu _a (r)$, and the optical fluence, $\varphi (r,\mu^{\prime}_t)$, which is the optical energy per unit area reaching each point of the sample volume. According to the Beer-Lambert law, the optical fluence (or intensity), depends on the incident fluence $\varphi _i(x,y)$, (transversal profile in the *z* direction) and is exponentially attenuated as the light propagates into the medium. This attenuation is generally governed by both absorption and scattering interactions and is characterized by the transport interaction or extinction coefficient as $\mu^{\prime}_t(r) = \mu _a(r) + \mu^{\prime}_s(r)$. Here, the medium scattering is characterized by the reduced scattering coefficient, $\mu^{\prime}_s = \mu _s(1-g)$, considering the forward scattering of the light beam propagating in one direction (*z*), which is the the isotropic scattering coefficient, $\mu _s$, modulated by the anisotropy factor (*g*). These characteristic light-matter interaction coefficients all have inverse distance units ([$\hbox {m}^{-1}$]) and are interpreted as the mean free path of the light propagation within the medium. Please also note that they generally depend on wavelength $\lambda$ and position *r*, although this is not explicitly indicated for simplicity in the above and further expressions.

#### Photoacoustic measured signal and integrated pressure model

For all the experimental tests, the measurement of the PA signal (as outlined in previous sections) is performed as the PA average signal, $\overline{p}^m_0$. This is obtained by summing the initial pressure amplitudes, ${p}_0(n)$, measured from the reconstructed image pixels of the cannula container cross section and dividing by the total number of pixels, *N*, which reads as

2 $$\begin{aligned} \overline{p}^m_0 = \frac{1}{N} \sum _{n=1}^{N} {p}_{0}(n). \end{aligned}$$

This PA average signal measured as in Eq. (2) in Volts units can be related to the average pressure, $\overline{p}_0$, in Pascals ([N/$\hbox {m}^2$] or [J/$\hbox {m}^3$]) through the calibrated probe’s sensitivity, *S* ([V/Pa]), as $\overline{p}^m_0 = S \overline{p}_0$.

Therefore, to obtain the equivalent theoretical model of the average pressure, $\overline{p}_0$, the pressure spatial distribution of Eq. (1) must be integrated over the light absorbing volume *V* as

3 $$\begin{aligned} \overline{p}_0 = \frac{1}{V} \int _V p_0 (r) dr = \frac{\Gamma \eta _{th}}{V} \int _A \varphi _i (x,y) dS \int _0^l \mu _a \exp (-\mu^{\prime}_t z) dz \end{aligned}$$

Here, the first integral represents the transversal fluence $\varphi _i (x,y)$ over the area of incidence *A*, which, dividing by the sample volume *V*, yields the average optical energy density delivered to the sample, $\overline{E_i}$. This incident energy is also the average incident fluence over the optical path length *l*, so it can be written as, $\overline{E_i} = \overline{\varphi _i}/l$ [J/$\hbox {m}^3$]. The second integral then represents the exponential attenuation of the incident energy, which occurs along the sample length in the *z* direction and accounts for the absorption and scattering events using the extinction coefficient ($\mu^{\prime}_t$). Simultaneously, the absorption coefficient within the integrand ($\mu _a$) modulates the exponential decay since only the absorbed light contributes to the initial pressure and PA signal generation. A closed-form integral solution of Eq. (4) can be easily found for constant coefficients if the extinction and absorption coefficients are taken as the concentration-averaged values, $\mu \equiv \overline{\mu (r)}$. Since both averaged absorption and extinction coefficients are directly proportional to the sample particle concentration (as detailed further on), the integrated-model solution holds for general non-uniform concentrations within the sample because the average of any distribution is always constant provided that this volume contains the overall mass, $m_C$, of the concentration, $\rho = m_C/V$.

Consequently, the average pressure of the laser-excited volume is given as a function of the absorption and extinction coefficients as

4 $$\begin{aligned} \overline{p}_0 (\mu _a, \mu^{\prime}_t, l) = \Gamma \eta _{th} \overline{E_i} \left( \frac{\mu _a}{\mu^{\prime}_t}\right) \left[ 1 - \exp (-\mu^{\prime}_t l)\right] \eqsim \Gamma \eta _{th} \overline{\varphi _i} \mu _a = \Gamma \eta _{th} \overline{E_i} A. \end{aligned}$$

This relation essentially models the inherently nonlinear saturation behaviour of the laser-excited average pressure for large values of the product $\mu^{\prime}_t l\gg 1$, which is just the total attenuation along the sample length, $A_l\equiv \mu^{\prime}_t l$. In this saturation regime, $\overline{p}_0$ asymptotically approaches its maximum pressure value, $\Gamma \eta _{th} \overline{E_i} (\mu _a/\mu^{\prime}_t)$, implying that all the incident optical energy is attenuated within the sample. As expected, the fraction $\mu _a/(\mu _a + \mu^{\prime}_s)$ represents the reduction of the overall pressure level produced by the scattered light, and only the absorbed light will eventually contribute to the pressure generation. Conversely, the linear regime occurs for samples with a small product $\mu^{\prime}_t l<1$, or attenuation $A_l<1$, and the exponential decay of Eq. (4) is approximated on the right-hand side of this equation using the first-order term of a Taylor series expansion. Since the extinction coefficient cancels out in the linear approximation, the average pressure is only proportional to the absorbed optical energy, and, is not influenced by the scattered light in this regime, for which the attenuation defined as the usual absorbance as, $A_l\equiv \mu _a l$.

#### Photoacoustic integrated pressure in terms of molar and mass concentration

The equivalent model expression of Eq. (4) in terms of the molar, *C* ([mol/mL]), and mass concentrations, $\rho$ ([mg/mL]), which are useful for experimental measurements, can be directly obtained by substituting the corresponding absorption and extinction coefficients. These are directly related to the concentration-normalized coefficients by the well-known relation, $\mu = k\epsilon C = k\beta \rho$. Here, the factor *k* is introduced for the decadic attenuation of the Beer-Lambert law for liquids ($k=\mathrm {ln(10)}$, used in our experiments), instead of the pure exponential decay for gases ($k=1$). The molar coefficients are $\epsilon _a$, $\epsilon^{\prime}_t$ ([$\hbox {cm}^{-1}$ $\hbox {mol}^{-1}$mL]), and the mass-specific coefficients are $\beta = \epsilon /M_w$ ([$\hbox {cm}^{-1}$ $\hbox {mg}^{-1}$mL]), knowing that both kinds of sample concentrations are related through the molecular weight of the absorber, $M_w$ ([mg/mol]), as $\rho =CM_w$. Therefore, the integrated pressure model as a function of the average sample density ($\rho$) and the optical path length (*l*) is finally written as

5 $$\begin{aligned} \overline{p}_0 (\rho ,l) = \Gamma \eta _{th} \overline{E_i} \left( \frac{\beta _a}{\beta^{\prime}_t}\right) \left[ 1-\exp (-k\beta^{\prime}_t \rho l) \right] \eqsim \Gamma \eta _{th} \overline{\varphi _i} k\beta _a \rho , \end{aligned}$$

where the right-hand term shows the linear regime relation of the average pressure varying with the average mass concentration or sample density. As before, the linear approximation is obtained for the small attenuation condition, $A\equiv \mu^{\prime}_t l<1$, which is now equivalent to $k\epsilon^{\prime}_t C l = k \beta^{\prime}_t \rho l<1$. Note that the ratios of different absorption and extinction coefficients are the same due to their proportionality ($\mu _a/\mu^{\prime}_t = \epsilon _a/\epsilon^{\prime}_t = \beta _a/\beta^{\prime}_t$).

#### Linear regime approximation for experiments

The linear fitting relation used for in our experiments involves the averaged values of pressure with respect to the mass concentration, and is rewritten from Eq. (5) as

6 $$\begin{aligned} \overline{p}_0 (\rho ) = \alpha _1 \rho + \alpha _2. \end{aligned}$$

In this expression, the slope factor $\alpha _1 = \Gamma \eta _{th} \overline{\varphi _i} (\mu _a/\rho ) = \Gamma \eta _{th} \overline{\varphi _i} k\beta _a$, with physical units of energy divided by mass ([mJ/mg]), indicates that a greater slope corresponds to a higher absorption within the sample at constant fluence. Moreover, $\alpha _2$ is a constant added to account for the baseline or background pressure signal of sample dilution. Interestingly, the limit of concentration (density) allowed in a given sample length to behave linearly is,

7 $$\begin{aligned} \rho < \frac{\Gamma \eta _{th} \overline{\varphi _i}}{\alpha _1l_s} \end{aligned}$$

which is directly derived from the linear approximation condition, $\mu^{\prime}_t< \mu _a < 1/l_s$ applied to the slope factor ($\alpha _1$) for a sample length ($l = l_s$). Additionally, for experimental measurements, the PA signal is related to the average integrated pressure by the probe’s sensitivity (in [V/Pa] units) as, $\overline{p}^m_0 (\rho ) = S \overline{p}_0(\rho )$, and the slope factor is $\alpha _1^m = S \alpha _1$ ([$\hbox {Vmg}^{-1}$mL)]). From this, if the probe’s sensitivity is known, the mass concentration limit of the linear regime (Eq. (7)) can be calculated from $\rho < S(\Gamma \eta _{th} \overline{\varphi _i} / \alpha ^m_1 l_s)$.

### Cytotoxicity test

The cytotoxic effects of the covalent organic frameworks (COFs) LZU-1 and TAPB-PDA were evaluated using MTS viability assays (3-(4,5-dimethylthiazol-2-yl)-5-(3-carboxymethoxyphenyl)-2-(4-sulfophenyl)-2H-tetrazolium) in NIH/3T3 (mouse fibroblast) and HeLa (human cervical cancer) cell lines. Both cell lines were purchased from the American Type Culture Collection (ATCC, Rockville, MD). Cells were seeded at a density of 4,000 cells per well in a 96-well plate and cultured in 5% CO2 at 37 $^\circ$C for 24 h. Then, cells were treated with LZU-1 and TAPB-PDA materials with doses ranging from 0.5 to 100 $\mu$g/mL during 24 h. Following the incubation period, 10 $\mu$L of MTS solution was added to each well and incubated for another 3.5 h. Absorbance was measured at 490 nm using a BioTek Synergy H1 Multimode Reader. Three independent experiments were performed for every sample with each experiment conducted in triplicate.

## Results

### Preparation of stable nanoparticles with high PA response

**Figure 2:**
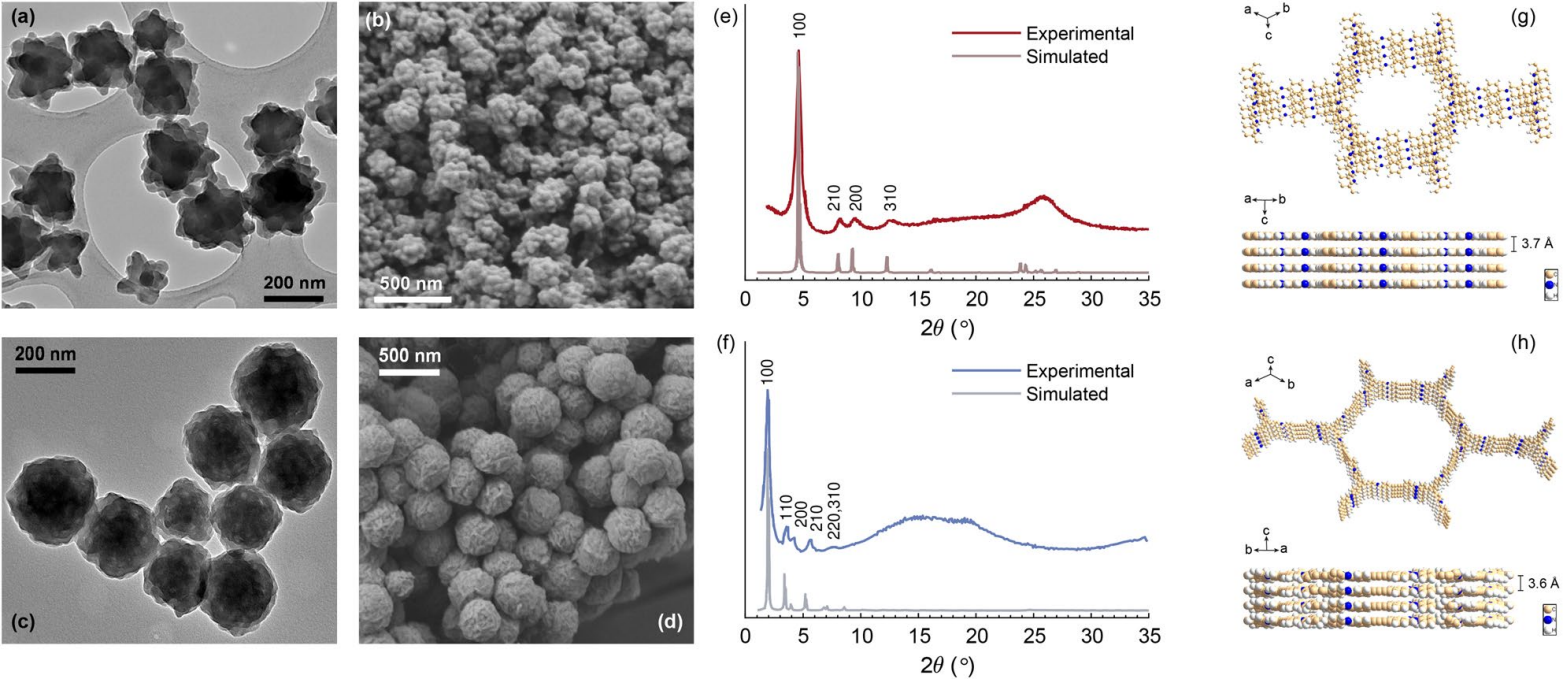
Characterization of LZU-1 (above) and TAPB-PDA (below) nanoparticles. (**a**,**c**) TEM images. (**b**,**d**) FESEM images. (**e**,**f**) Powder XRD patterns of as-prepared materials (red/blue line) and simulated pattern for an eclipsed structure (grey lines). (**g**,**h**) Simulated 2D eclipsed layered-sheet arrangement indicating the interlayer space. Molecular structures were depicted with Diamond 3.2 from Crystal Impact software.

The two types of nCOFs, TAPB-PDA and LZU-1, were synthesized using carefully optimized methods, tailored to each material^25,26^. This approach enabled precise control over reaction parameters, including solvents selection, catalysts choice, temperature, and reaction time, to ensure the formation of homogeneous and highly crystalline nanoparticles. Solvent mixtures were chosen to maximize precursor solubility and promote uniform nucleation, while catalysts facilitated the condensation of functional groups, thereby ensuring the formation of stable imine bonds and well-defined crystalline structures. These methods produced reproducible nanoparticles, as validated by DLS and XRD analyses, which demonstrated minimal variability in particle size and crystallinity across multiple synthesis batches. TEM and field-emission scanning electron microscopy (FESEM) images (Fig. 2a–d), reveal that the synthesized LZU-1 and TAPB-PDA nanoparticles exhibit good dispersion (DLS polydispersity index <0.2). LZU-1 nanoparticles display a star-shaped morphology with an average diameter of approximately 255 nm (DLS measurements, Supplementary Fig. S1) whereas TAPB-PDA adopts a spherical morphology with an average size of 395 nm. Powder XRD analysis confirmed the high crystallinity of both imine-linked nCOFs, revealing hexagonal symmetry and reflections corresponding to a two-dimensional eclipsed layered-sheet structure (Fig. 2e,f), with lattice parameters for LZU-1 of a = b = 22.0 Å and c = 3.7 Å and of a = b = 52.0 Å and c = 3.6 Å for TAPB-PDA (Fig. 2g,h). The porosity and surface areas of TAPB-PDA and LZU-1 were measured by adsorption-desorption N2 isotherms, matching a standard type IV, and the application of the BET model yielded surface areas of 215.2 and 257.9 m^2^ g^-1^, respectively (Fig. S2a–b and Table S1). DRS analysis indicated that both materials exhibited tail absorption at 532 nm. TGA reports clearly demonstrated the excellent thermal resistance and structure stability of these nCOFs. There was no incipient decomposition below 350 $^{\circ }$C (see Supplementary Fig. S3). In addition, FTIR analysis confirmed the synthesis of imine-linked COFs. A broad band observed at 3300–3450 cm^-1^ corresponds to the stretching vibration of terminal N-H groups, while the band at 1680–1690 cm^-1^ belongs to terminal C=O stretching vibration. Moreover, the peak at 1618–1620 cm^-1^ is unequivocally due to the C=N stretching vibration, indicating the formation of the imine structure (see Supplementary Fig. S4).

MSN-ATTO532 nanoparticles were selected as the reference material due to their comparable size and structural characteristics to the COFs under investigation. These nanoparticles were synthesized as spheroidal monodispersed nanoparticles (Supplementary Fig. S5) with an average diameter of 190 nm, as determined by TEM measurements over 250 particles. However, DLS measurements indicated a slightly larger hydrodynamic average diameter of 255 nm (Supplementary Fig. S1), attributed to the partial formation of dimers and trimers in aqueous medium. The powder XRD measurement showed the typical pattern of a nanoscale MCM-41 phase (Supplementary Fig. S7)^27^. Consistently, MSN-ATTO532 exhibited type IV N2 adsorption isotherms (Supplementary Fig. S2c), with BET specific surface area of 569.8 m^2^g^-1^. DRS analysis indicated a distinct absorption maximum at 532 nm, characteristic of ATTO532. Finally, the amount of ATTO532 conjugated on the MSN-NH2 surface was quantified by elemental analysis, revealing an ATTO532 loading of 0.025 – 0.04 mmol/g^-1^.

### Photoacoustic validation of nCOFs

**Figure 3:**
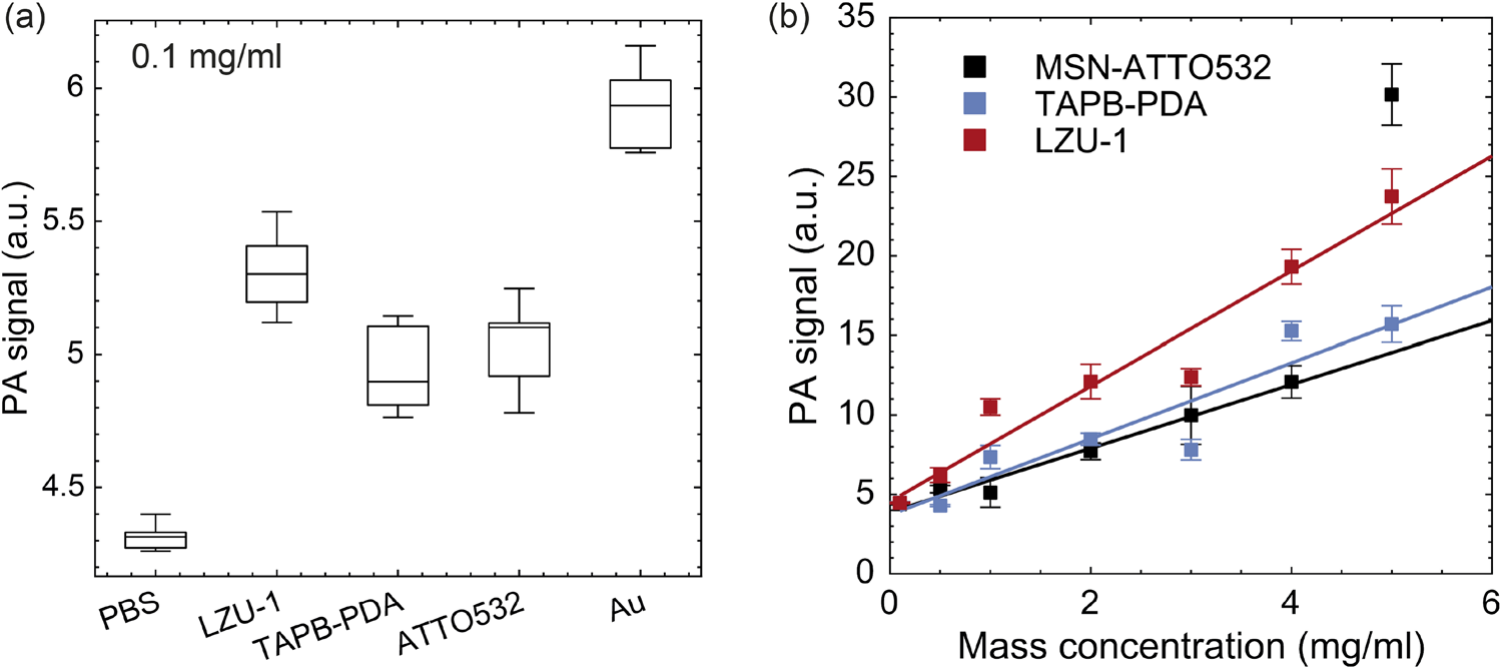
Photoacoustic response of nCOFs. (**a**) PA response of gold nanoparticles (commercial reference) in comparison to nCOFs (TAPB-PDA and LZU-1) as well as MSNs labelled with the ATTO532 fluorochrome, all measured at a concentration of 0.1 mg/mL. The PBS medium, in which the samples are dissolved, serves as the baseline PA response. (**b**) PA response as a function of increasing nanoparticle concentration obtained experimentally (square markers), with data fitted using the linearized model (continuous lines).

**Figure 4:**
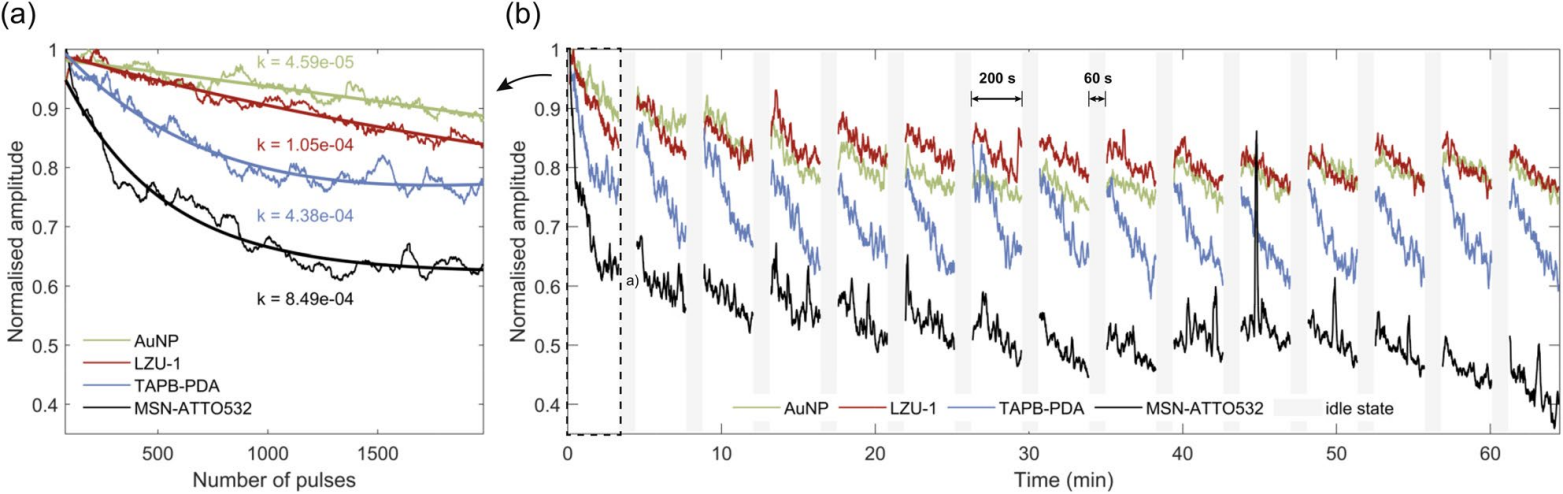
Photostability. (**a**) Normalized PA amplitude recorded during the first excitation set of 2,020 pulses for AuNPs, LZU-1, TAPB-PDA and MSN-ATTO532. Solid curves show the double-exponential fits used to determine the photobleaching rate *k*. (**b**) Normalized PA amplitude over 15 consecutive excitation sets separated by 60 s idle intervals.

As shown in Fig. 3a, nCOFs samples diluted to concentration of 0.1 mg/mL (the maximum concentration available for AuNPs), and excited at laser wavelength of 532 nm, generate PA responses of the same order of magnitude as AuNPs. Each sample was evaluated in six. Box-whisker plots show the median, indicated by the central horizontal line, the lower and upper edges of the box correspond to the 25th and 75th percentiles, and the whiskers extend to the most extreme data points that do not qualify as outliers. No outliers were detected in these measurements. The strong PA response observed for MSN-ATTO532, which serves as a reference, is expected, given that the grafted fluorochrome exhibits maximal absorption at the selected laser wavelength. Interestingly, despite being far from their maximal absorption at this wavelength, as shown in the optical spectra of Fig. 4a, both TAPB-PDA and LZU-1 materials present significant PA signal amplitudes. This behaviour can be attributed to the strong $\pi$-$\pi$ interaction among the layers of these imine COFs, which enables absorption along a wide wavelength range and the releasing of vibrational energy to produce the PA signal^63^. Assuming an eclipsed structure for both materials, as confirmed by powder XRD patterns, the close layer packing (3.6 Å for TAPB-PDA and 3.7 Å for LZU-1) is consistent with higher $\pi$-$\pi$ stacking energy^64^.

Figure 3b illustrates the trend of the PA response for the nCOFs and ATTO532-labelled MSNs as their concentration increases up to 5 mg/mL. A total of 24 replicates per sample were measured and averaged for each concentration. The experimental data were fitted using the linear relation of Eq. (6) with the fitting parameters $\alpha _1$, the PA signal variation slope with nanoparticle concentration, and $\alpha _2$, the background level of the PBS solution. The linear regression results of the experimental data show strong correlation, with R-squared values of 0.98, 0.88 and 0.94, respectively for MSN-ATTO532, TAPB-PDA and LZU-1, indicating that nanoparticle concentrations are in the linear regime of the full model of Eq. (5). The corresponding linear factors, $\alpha ^m_1 =$ 2.00, 2.38 and 3.59, where the larger slope means the higher absorption of the LZU-1 (red), and a common PBS background signal of $\alpha _2 = 4.5$. Additionally, from the linear regime condition and for a fixed cannula diameter ($l_s = 1$ mm), the maximum average absorption coefficient for the evaluated nanoparticle concentrations can be estimated as $\mu^{\prime}_t< \mu _a < 10$ cm^-1^. Therefore, the high correlation of the experimental data confirms the linear regime of the full model described by Eq. 5, suggesting that the nonlinear saturation of the PA signal may occur at higher sample concentrations. However, this behaviour could not be observed up to the maximum tested concentration of 5 mg/mL.

The MSN-ATTO532 data were fitted up to a concentration of 4 mg/mL, as the 5 mg/mL sample was identified as an outlier. This anomalously high value is attributable to strong nanoparticle aggregation of the samples, as shown in the TEM image in a high concentration suspension (see Supplementary Fig. S6), this aggregation produced nanoparticles deposition at the bottom of the cannula by gravity, as observed in the PA-US images of the MSN-ATTO532 samples in Fig. S13, and also in the sample photographs in Fig. S10, of the supplementary information. Then, the stronger effect of the 5 mg/mL concentration would lead to adding more nanoparticle’s mass into the cross-sectional window of the cannula used to calculate the integrated PA signal, which may come from other non-imaged sections. This would therefore mean a significant elevation of the average concentration value and the resulting PA signal outlier. Notably, the PA signal response would correspond to a concentration of $\rho \eqsim 13$ mg/mL by extrapolating the linear fitting Eq. (6) of MSN-ATTO532 samples data, more than doubling the supposed 5 mg/mL average concentration.

### Photostability

The photostability of the four contrast agents, AuNPs, LZU-1, TAPB-PDA, and MSN-ATTO532, was systematically investigated under prolonged pulsed laser excitation at 532 nm. Figure 4a shows the PA signal amplitude evolution during continuous laser irradiation, where all agents were exposed to a sequence of 2,020 laser pulses. The normalized PA amplitude decreased progressively for all samples, indicating a photothermal bleaching process. The photothermal bleaching rate, *k*, was extracted from a double-exponential fitting of the decay curves (see section Methods).

The lowest photobleaching rate was measured for AuNPs ($k = 4.59 \times 10^{-5}$), consistent with their high optical and thermal stability under excitation at 532 nm. This result is expected given that spherical AuNPs exhibit a strong localized surface plasmon resonance (LSPR) near this wavelength, allowing efficient photothermal conversion without significant structural degradation. It is worth noting, however, that this stability is highly dependent on excitation wavelength; Au nanorods and other anisotropic Au nanostructures absorb efficiently in the near-infrared (NIR) range, but undergo rapid reshaping to spheres under prolonged irradiation, leading to a marked loss of NIR absorbance^65^. LZU-1 and TAPB-PDA, the two covalent organic frameworks (COFs), exhibited good photostability with intermediate photobleaching rates ($k = 1.05 \times 10^{-4}$ and $k = 4.38 \times 10^{-4}$, respectively). This behaviour is consistent with their molecular nature, as COFs share similarities with semiconducting polymer nanoparticles^66^. Notably, the photobleaching observed in these COFs may be partially reversible, as indicated by their dynamic response under intermittent irradiation. MSN-ATTO532 showed the highest photobleaching rate ($k = 8.49 \times 10^{-4}$), with a sharp decline in signal even within the first few hundred pulses. This is consistent with the known instability of fluorophores such as ATTO532 under repetitive excitation. Since the photoacoustic signal in this case arises almost exclusively from the embedded dye, the degradation kinetics closely resemble those of the free molecule in solution^67^.

The behavior of the contrast agents under an intermittent excitation protocol, shown in Fig. 4b, provides additional insight into potential recovery mechanisms. Here, 15 excitation sets were separated by 60 s idle periods, allowing the samples to cool and partially recover between irradiations. In this context, the COFs LZU-1 and TAPB-PDA exhibited strong signal recovery after each idle phase, suggesting that the photobleaching mechanisms involved include reversible components. These may stem from various reasons, including transient excited states, reversible structural reorganizations, or localized thermal effects, as the off periods allow dissipation of accumulated heat, potentially reversing thermally induced conformational or electronic changes within the framework structure. In contrast, AuNPs showed an inferior recovery, with a more stable signal across excitation cycles, consistent with their photostability. Meanwhile, MSN-ATTO532 did not benefit from the idle periods, confirming that its photodegradation is dominated by irreversible molecular processes.

Overall, the obtained results for AuNPs and MSN-ATTO532 are in line with reported studies^66–68^, with slight differences likely associated with the higher irradiated energy levels employed in this work, with the former offering excellent photostability under 532 nm and the latter degrading rapidly, as expected. The COFs, although more susceptible to photothermal bleaching than AuNPs, offer good photostability, especially considering the high energy levels employed in this work, and show partial reversibility under pulsed conditions, which may be advantageous in imaging protocols involving intermittent or low-duty-cycle illumination.

### NIR photoacoustic spectroscopy

**Figure 5:**
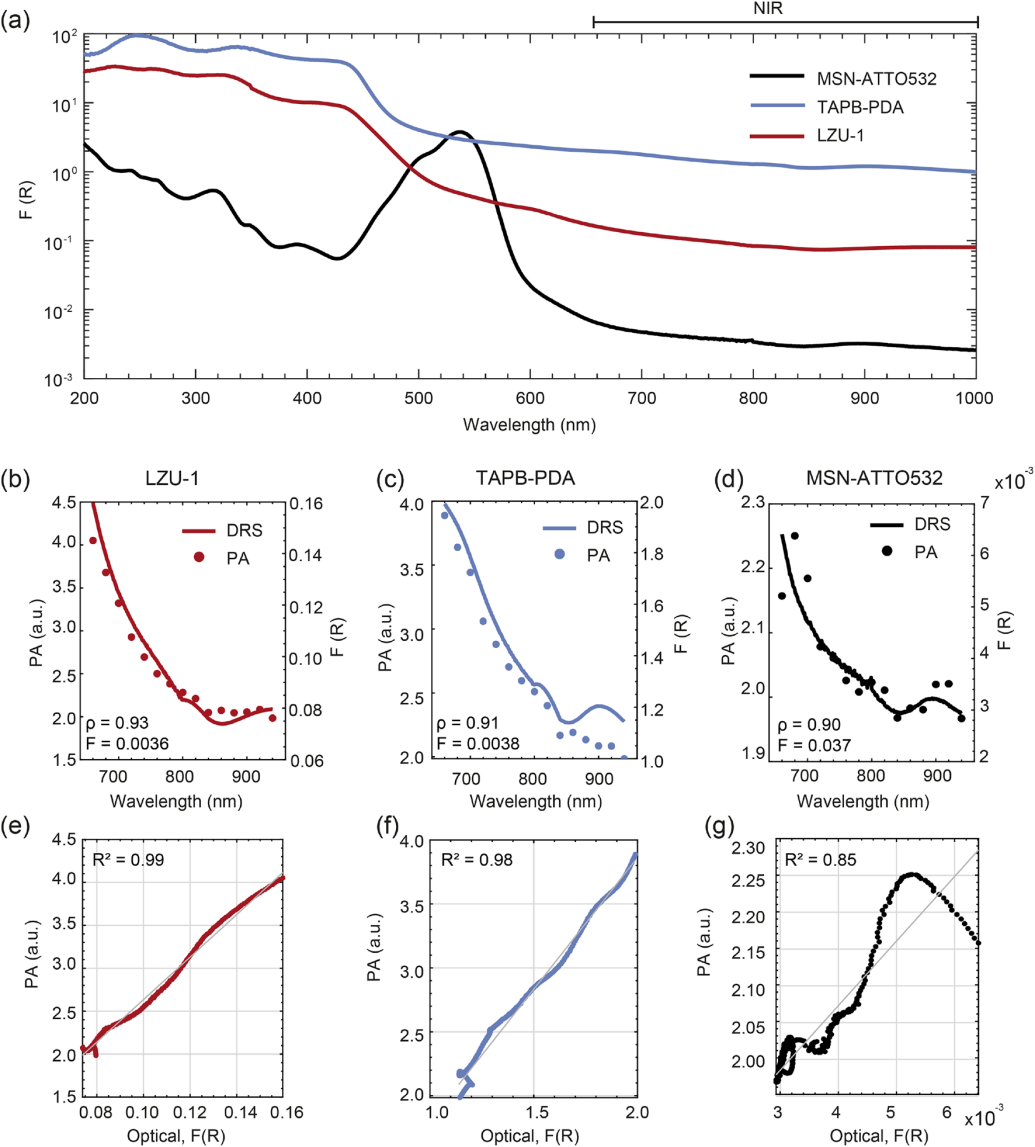
NIR spectroscopy. (**a**) Optical absorption spectra of LZU-1, TAPB-PDA, and MSN-ATTO532 nanoparticles. (**b**–**d**) Overlap of the PA and NIR optical absorption spectra in the range of 660-940 nm (**e**–**g**) Scatter plot comparing optical absorption with PA signal, accompanied by linear regressions (grey line) to assess the similarity of the spectral curves, with R^2^ as score.

To assess the potential of the proposed nCOFs within the NIR window, the PA response of the two nCOFs, along with MSN-ATTO532 nanoparticles, was evaluated at a concentration of 0.1 mg/ml, across a wavelength range from 660 to 940 nm at 20 nm increments. Figure 5a presents the optical absorption spectrum from 200 to 1000 nm obtained through DRS measurements. These spectra, acquired using a commercial spectrophotometer, demonstrate that both types of nCOFs exhibit a broad absorption band spanning approximately from 200 to 500 nm, with TAPB-PDA displaying a higher absorbance. Conversely, MSN-ATTO532 exhibits a narrowband absorption peak with absorbance values comparable to those of TAPB-PDA, despite the latter exhibiting a tail absorption at this wavelength range. Although both TAPB-PDA and LZU-1 maximal absorption peaks are located outside the NIR window, the measured PA signals were sufficiently strong to generate images and compute the integrated PA amplitude within the cannula, as detailed in the experimental methods.

Figure 5b–d presents a comparison between DRS measurements (solid lines) and the photoacoustic spectra (circular markers) for the TAPB-PDA, LZU-1 and MSN-ATTO532 nanoparticles, respectively. The data points for the PA spectra were obtained as the average of six measurement replicates. It is important to note that amplitude values are labelled separately for the photoacoustic (left axes) and DRS measurements (right axes) to facilitate a qualitative comparison. The results demonstrate that both spectra exhibit a similar decreasing absorption profile with increasing wavelength across the NIR window. As shown in the figure, the optical absorption levels for the nCOFs (TAPB-PDA and LZU-1) are higher than those observed for the MSN-ATTO532 nanoparticles.

To quantitatively compare the similarity between the optical and PA spectral curves, a linear regression analysis was performed on the scatter plot, with optical data points plotted on the x-axis and PA experimental data on the y-axis, as shown in Fig. 5e–g. The coefficient of determination, $R^2$, was used to evaluate the goodness of fit, providing a measure of the similarity between the two spectral curves. The $R^2$ values obtained are 0.99, 0.98 and 0.85 for LZU-1, TAPB-PDA and MSN-ATTO532, respectively. These results suggest that the optical and PA spectral curves of MSN-ATTO532 are more dissimilar than those of the two types of nCOFs, as qualitatively observed in Fig. 5b–d.

### Cytotoxicity test

**Figure 6:**
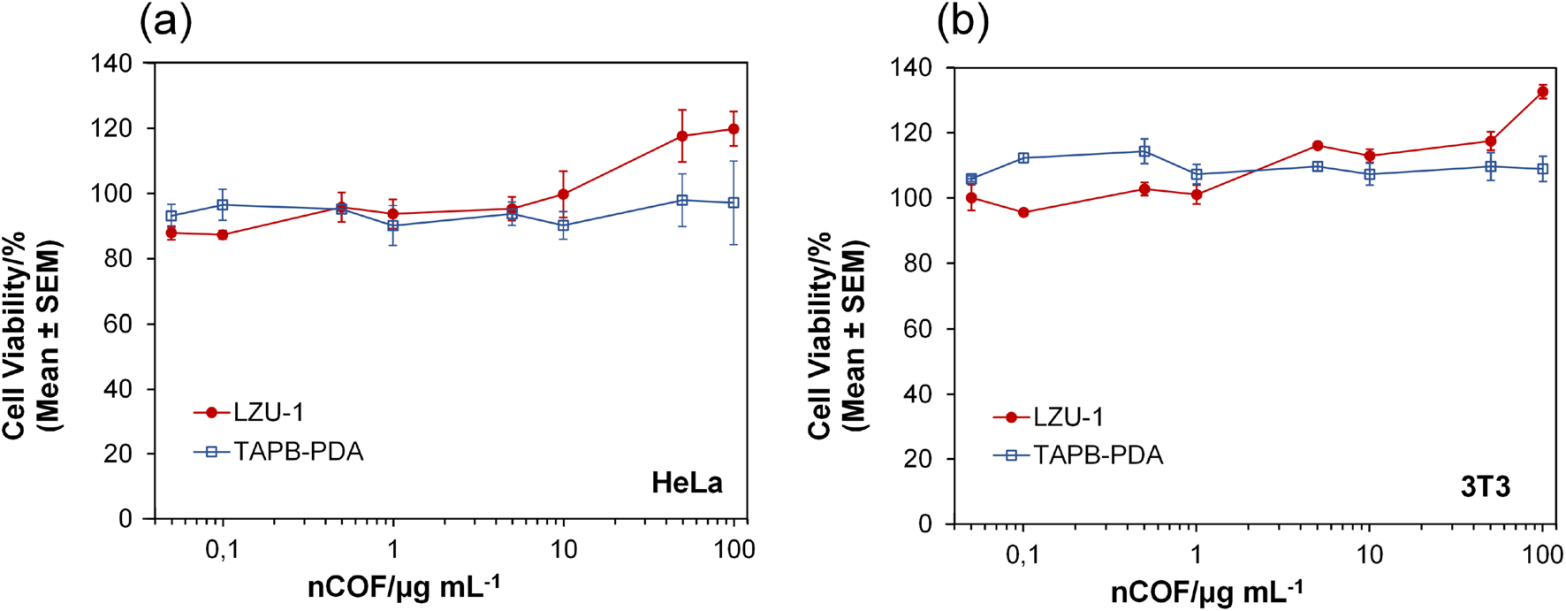
Citotoxicity test. In vitro MTS cell viability assays in HeLa (**a**) and 3T3 (**b**) cell lines after incubation with LZU-1 and TAPB-PDA covalent organic frameworks. All samples were incubated for 24 h. Each value represents the mean ±standard error (SEM) of three independent experiments.

The in vitro cytotoxicity assays, shown in Fig. 6, revealed that both LZU-1 and TAPB-PDA nanoparticles exhibit excellent biocompatibility, with cell viability exceeding 85 % at all tested concentrations. These findings are consistent with previous studies that report minimal cytotoxic effects of covalent organic frameworks (COFs) in similar cell models^69^.

The purely organic composition of LZU-1 and TAPB-PDA, coupled with the absence of heavy metals, likely contributes to their low toxicity, distinguishing them from conventional inorganic nanoparticles such as gold or silica-based systems. This unique feature underscores the potential of these materials for safe in vivo applications, particularly at higher concentrations, where inorganic nanoparticles often exhibit increased toxicity due to accumulation and oxidative stress. Additionally, the biodegradability of COFs, including LZU-1 and TAPB-PDA, has also been demonstrated in studies showing their gradual degradation under physiological conditions. This process is facilitated by hydrolysis of imine bonds in acidic or enzymatic environments, a hallmark feature of imine-based COFs^70^. Furthermore, the structural flexibility and organic nature of these COFs provide further advantages for biomedical applications. Previous studies have highlighted their potential for drug delivery, wound healing, and other therapeutic uses, owing to their biocompatible profile and ability to degrade safely in biological environments^25^. Taken together, these findings underscore the biocompatibility and biodegradability of LZU-1 and TAPB-PDA as critical attributes for their utilization in photoacoustic imaging and other advanced biomedical applications.

## Discussion

The results shown in this work demonstrate that both LZU-1 and TAPB-PDA nanoparticles exhibit significant potential as exogenous contrasts in photoacoustic imaging. At low concentrations (e.g., 0.1 mg/mL), their response is comparable to that of gold nanoparticles (AuNP), although the working wavelength of 532 nm corresponds to the tail absorption region of the nCOFs. More importantly, these nCOFs offer notable advantages over inorganic functionalized nanoparticles (e.g., MSN-ATTO532), primarily due to their highly biocompatible profiles and the absence of leaching of active molecules. Additionally, these nanoparticles are fully biodegradable, with natural elimination routes, primarily through renal and biliary excretion^71^.

Furthermore, as non-porphyrin-based nCOFs, TAPB-PDA and LZU-1 provide additional biological safety^57,72^. Their lower toxicity profile enables the administration of higher doses of the contrast agent without posing potential harm to the patient. This is particularly advantageous, as the PA response, and consequently, image contrast of nCOFs can be significantly enhanced by increasing their concentration, as well as enabling deeper penetration into tissues, as demonstrated in Fig. 3b, constituting a substantial advantage over other exogenous contrast agents. In the experimental regime explored in this study, a linear relationship was observed between the average PA pressure and nCOFs concentration^73^.

The obtained results also demonstrate the feasibility of utilizing these nCOFs in the NIR window. Not only do they provide detectable signals even at low concentrations (0.1 mg/mL), but, as previously noted, the signal amplitude can be notably enhanced by increasing the nanoparticle concentration. In this regard, the use of nCOFs offers two key advantages. First, since endogenous chromophores, such as haemoglobin, exhibit a reduced light absorption in the NIR window, the proposed nCOFs can be employed to achieve high-contrast photoacoustic imaging without interference from other endogenous contrast agents. Additionally, the reduced light scattering characteristic of the NIR window can enhance photoacoustic imaging penetration, further improving imaging capabilities.

One of the main challenges in the engineering of COF structures is shifting their absorbance spectrum into the NIR region, thereby enabling their use in in vivo photothermal and photoacoustic applications^53,74^. In this context, there are three possible pathways to reach this target: (i) the introduction of specific COF ligands showing absorbance in the NIR spectrum, with porphyrin-derivatives being the most commonly employed^27,52–56^. However, as previously discussed, this may lead to toxicity issues^57^; (ii) partial oxidation of COF ligands allow to achieve satisfactory NIR electrochromic properties in specific COF structures^75,76^; and (iii) the encapsulation and grafting of molecules with absorbance spectra within the NIR zone (e.g., indocyanine), a strategy widely applied to various materials, also possible in COFs^77–79^. However, this method is constrained by limited loading ability and the threat of leaching, which restricts its use in in vivo applications.

In this work we have targeted a distinct approach by exploring the potential of two-dimensional COF structures with closely packed layers and high $\pi$–$\pi$ stacking energy. This structural configuration enables absorption across a broad wavelength range and facilitates the release of vibrational energy, resulting in a significant PA signal amplitude^63^. The results demonstrate that LZU-1 and TAPB-PDA nCOFs can produce an enhanced PA response within the NIR window, despite operating within the residual region of their absorption spectrum. In layered materials, it is well known that $\Pi$–$\Pi$ stacking leads to aggregation, which can cause photoluminescence quenching due to non-radiative pathways^80^. Aggregation-caused quenching (ACQ) is a typical energy-consuming effect observed in fused aromatic compounds with planar structures. The excited state commonly decays to the ground state via non-radiative transitions, resulting in a rapid decrease in fluorescence intensity. In this context, 2D COF layers with abundant $\pi$–electrons assemble via $\pi$–$\pi$ stacking, and the interlayer distance is generally less than 4 Å, leading to tight packing structures and intense ACQ. In particular, 2D imine-linked COFs exhibit a significant decrease in luminescence intensity due to interlayer charge transfer^81^. Furthermore, a relationship has been demonstrated between fluorescence decrease by ACQ and photoacoustic generation efficiency in other materials^82^. Therefore, fluorescence loss by $\pi$–$\pi$ stacking in 2D COFs is a promising strategy for favoring non-radiative decay and enhancing the photoacoustic signal. In addition, as confirmed by powder XRD patterns, TAPB-PDA has closer layer packing than LZU-1 (3.6 Å for TAPB-PDA vs. 3.7 Å for LZU-1), which is consistent with higher $\pi$–$\pi$ stacking energy. This is probably the reason why TAPB-PDA sometimes outperforms LZU-1, for example resulting in a higher PA response in the NIR window. Furthermore, their high stability in water and favourable biocompatibility pattern position these nanoparticles as promising candidates for in vivo applications. In addition, the structural versatility of these nCOFs allows for functionalization with tumour-specific ligands or other targeting molecules, offering potential for precise biomedical imaging and theragnostics. Future research will focus on optimizing the optical properties of these nCOFs to improve their NIR absorption and photoacoustic performance. Moreover, comprehensive in vivo studies are essential to evaluate their biodistribution, clearance mechanisms, and long-term safety profiles. These efforts will be pivotal in advancing LZU-1 and TAPB-PDA nCOFs as next-generation contrast agents for molecular photoacoustic imaging, addressing current challenges in diagnostic and therapeutic imaging technologies. In this sense, due to the good biocompatibility of these materials observed in preliminary in vitro studies, an in vivo study of PA imaging, biodistribution and plasma clearance is currently being planned using a glioblastoma multiform (GBM) orthotopic rat model. The main objective of this study is to evaluate the ability of PAI to detect tumor margins, as this could provide valuable insights into the efficacy of targeted chemotherapy using 5-aminolevulinic acid as the targeting molecule, as previously demonstrated by our group^83^.

## Conclusion

In this study, non-porphyrin-based nano-covalent organic frameworks (nCOFs) were evaluated and experimentally validated as exogenous contrast agents for photoacoustic imaging. Two materials, LZU-1 and TAPB, were analysed and compared against established photoacoustic contrast agents, such as gold nanoparticles and mesoporous silica nanoparticles labelled with the ATTO532 chromophore, which served as baseline references. The results demonstrate that both LZU-1 and TAPB nCOFs produce a photoacoustic response comparable to that of the reference exogenous chromophores at their maximal absorption wavelength of 532 nm. Additionally, the porphyrin-free nature of these nCOFs, combined with their excellent biocompatibility and ease of clearance through natural pathways, makes their use at higher concentrations than inorganic exogenous contrast agents feasible. Consequently, by using larger concentrations (up to 5 mg/mL in this work) their photoacoustic response can be significantly enhanced, leading to improved image contrast and deeper tissue penetration. Furthermore, the potential of these nCOFs in the NIR window was demonstrated through experiments at wavelengths ranging from 660 to 940 nm, revealing PA amplitude levels superior to those of ATTO532-labelled MSN nanoparticles, even at the minimal concentration considered (0.1 mg/mL). These findings demonstrate the capacity of the proposed nCOFs to generate significant PA response across a broad wavelength range. Moreover, these organic materials can be easily functionalized with specific ligands for targeted applications, such as tumour-specific ligands for precise targeting. In addition, photostability assessments under repeated pulsed laser excitation revealed that both LZU-1 and TAPB-PDA exhibit good resistance to photobleaching and partial recovery under intermittent irradiation, reinforcing their suitability for sustained imaging protocols. In conclusion, owing to their enhanced photoacoustic performance, robust biocompatibility and photostability, and versatile design, these nCOFs hold great promise as contrast agents for in vivo photoacoustic imaging, advancing the potential for the clinical translation of molecular photoacoustic imaging technologies.

## Supplementary Information

Supplementary Information.

## Data Availability

Data used in this study will be provided by the corresponding author upon reasonable request.
