# Supplementary material for: Nanoparticles of non-porphyrinic covalent organic frameworks as contrast agents for photoacoustic imaging
Source: Sci Rep. 2025 Sep 30;15:33909. doi: 10.1038/s41598-025-08631-w (PMC12484929; doi:10.1038/s41598-025-08631-w)
Supplement: Supplementary file 1 — Supplementary Information. [file 41598_2025_8631_MOESM1_ESM.docx]

**Supplementary Information**

**Nanoparticles of non-porphyrinic covalent organic frameworks as contrast agents for photoacoustic imaging**

Irene Pi-Martín^a^, Carla Vidaurre-Agut^b^, Eva María. Rivero-Buceta^b^, Alejandro Cebrecos^a,^*, Juan José. García-Garrigós ^a,^*, Noé Jiménez^a^, José María Benlloch^a^, Pablo Botella^b,^*, Francisco Camarena^a*^

*^a^* Instituto de Instrumentación para Imagen Molecular (i3M), Universitat Politècnica de València-Consejo Superior de Investigaciones Científicas, Avenida de los Naranjos s/n, 46022 Valencia, Spain

*^b^* Instituto de Tecnología Química, Universitat Politècnica de València-Consejo Superior de Investigaciones Científicas, Avenida de los Naranjos s/n, 46022 Valencia, Spain

**1. Characterization of nanoparticles**

**1.1. Hydrodynamic diameter**

Physical-chemical characterization of nCOFs by Dynamic Light Scattering (DLS).

**Fig.** **S1**. Particle diameter (average and distribution) as determined by dynamic light scattering (DLS) measurement of as-synthesized nanoparticles.

**1.2. N_2_ adsorption-desorption isotherms**

**Fig. S2.** BET surface areas from the nitrogen adsorption-desorption isotherms at 77 K of as-synthesized materials. (a) LZU-1; (b) TAPB-PDA; (c) MSN-ATTO532.

**Table S1.** Main characteristics of materials tested in the present work.

| Sample | Particle diameter^a^  (nm) | Particle Diameter^b^  (nm) | Pore^c^  (nm) | Vp^c^  (cm^3^ g^-1^) | Area BET  (m^2^ g^-1^) |
| --- | --- | --- | --- | --- | --- |
| LZU-1 | 235.2 ± 29.2 | 255.2 ± 36.7 | 3.8 | 0.10 | 257.9 |
| TAPB-PDA | 378.6 ± 31.1 | 395.4 ± 46.3 | 3.8 | 0.20 | 215.2 |
| MSN-ATTO532 | 190.3 ± 67.5 | 255.3 ± 54.9 | 3.1 | 0.76 | 569.8 |

^a^Determined by TEM.

^b^Determined by DLS.

^c^Calculated from the N_2_ adsorption-desorption isotherms.

^d^Pore volume calculated from the N_2_ adsorption-desorption isotherms.

**1.3. Thermogravimetric analysis (TGA)**

**Fig. S3.** Thermogravimetric analysis of as-synthesized LZU-1 and TAPB-PDA samples.

**1.4. Fourier Transform Infrared (FTIR) spectroscopy**

**Fig. S4.** FTIR spectra of imine nCOFs obtained in the present work.

**1.5. Transmission Electronic Microscopy (TEM)**

**Fig. S5.** Transmission Electronic Microscopy (TEM) image obtained for as-synthesized MSN-ATTO532 particles.

**Fig. S6.** A TEM image of MSN-ATTO532 nanoparticles in a high-concentration suspension (5 mg/mL).

**1.6. Powder X-Ray Diffraction (XRD)**

**Fig. S7.** Powder X-Ray Diffraction (XRD) pattern obtained for as-synthesized MSN-ATTO532 particles.

**2. Photographs of the set of photoacoustic samples**


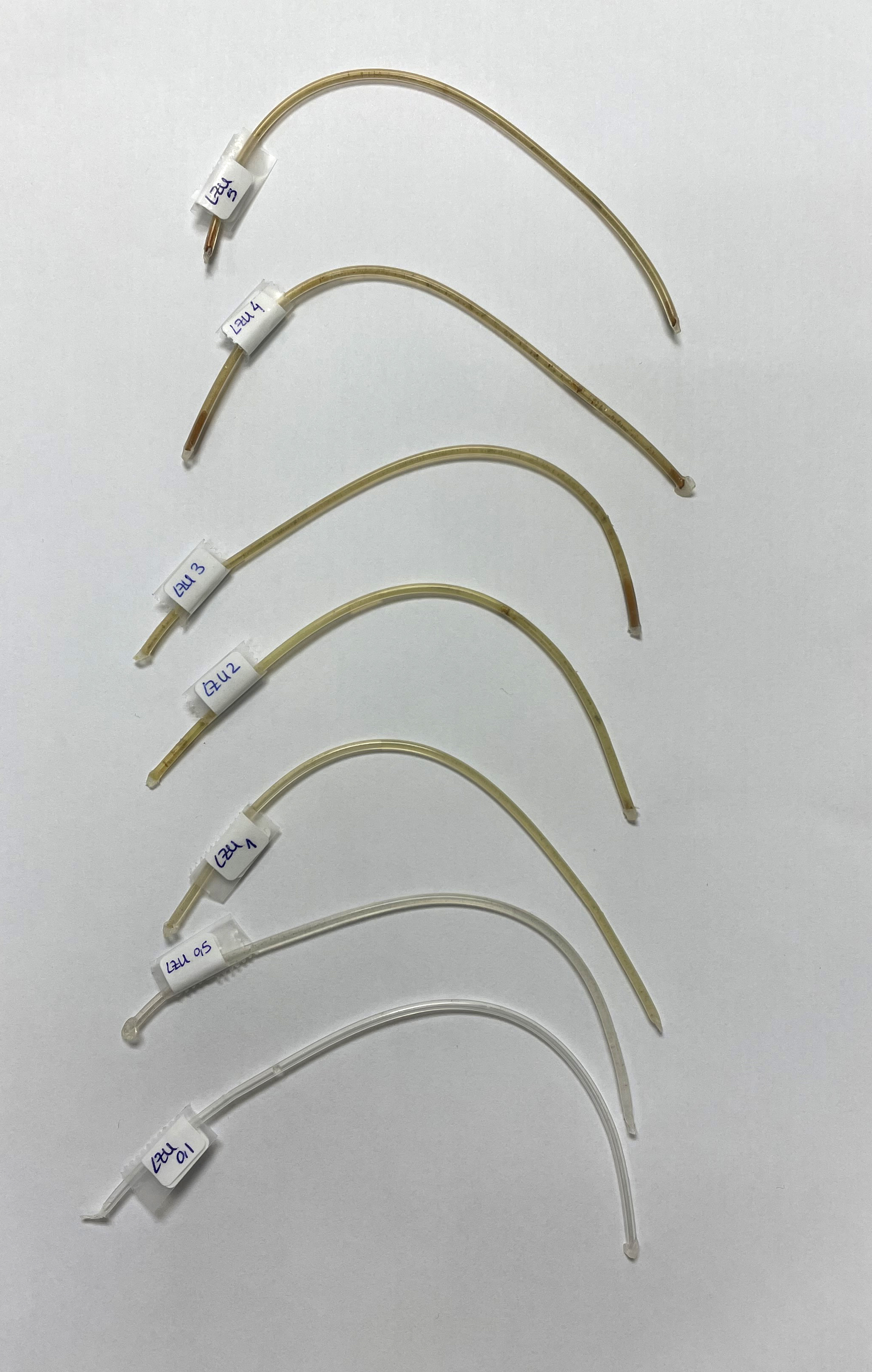


**Fig. S8.** Photograph of a sample for LZU cannulas for 0.1, 0.5, 1, 2, 3, 4, 5 mg/ml concentration.


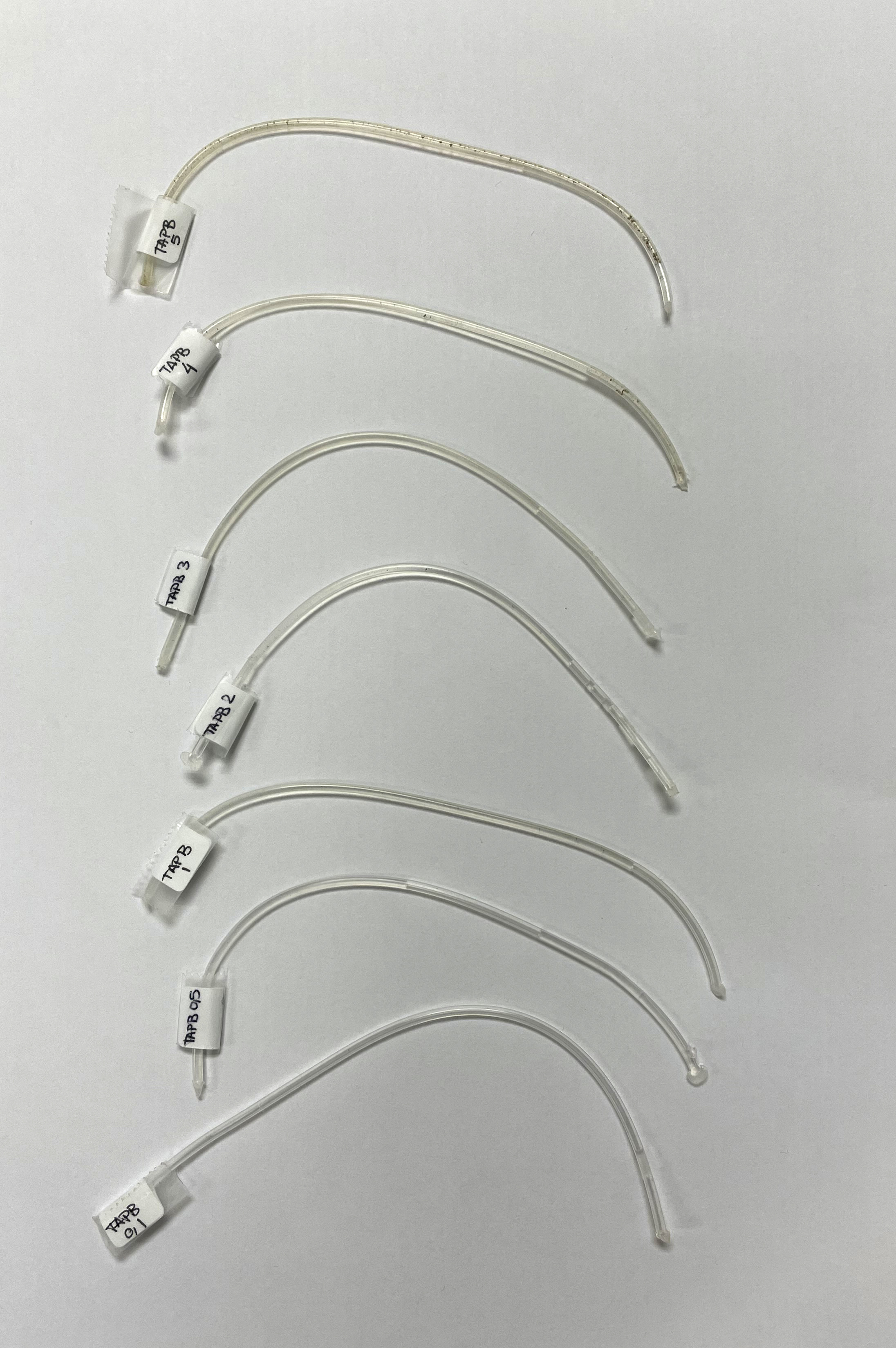


**Fig. S9.** Photograph of a sample for TAPB cannulas for 0.1, 0.5, 1, 2, 3, 4, 5 mg/ml concentration.


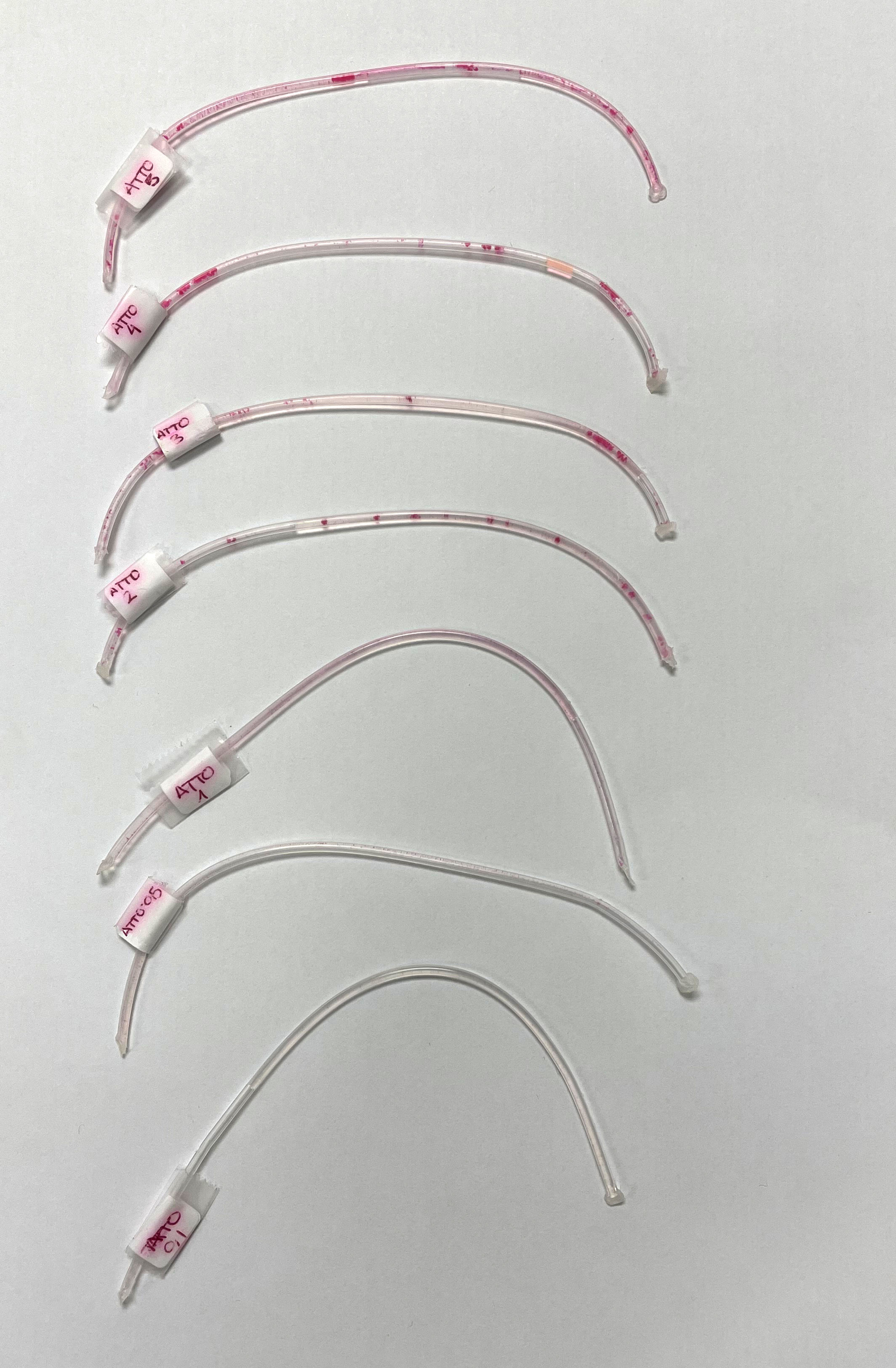


**Fig. S10.** Photograph of a sample for ATTO cannulas for 0.1, 0.5, 1, 2, 3, 4, 5 mg/ml concentration.

**3. Hybrid photoacoustic (color) and ultrasound images (gray) of the samples**


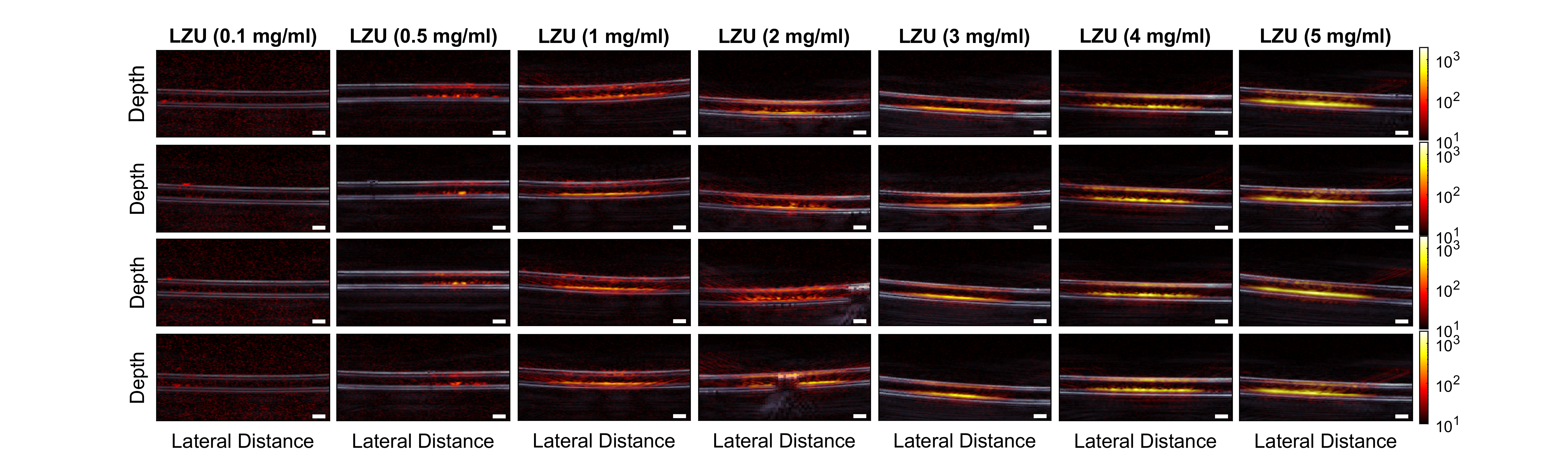


**Fig. S11.** Samples of fused imaged for LZU cannulas for 0.1, 0.5, 1, 2, 3, 4, 5 mg/ml concentration.


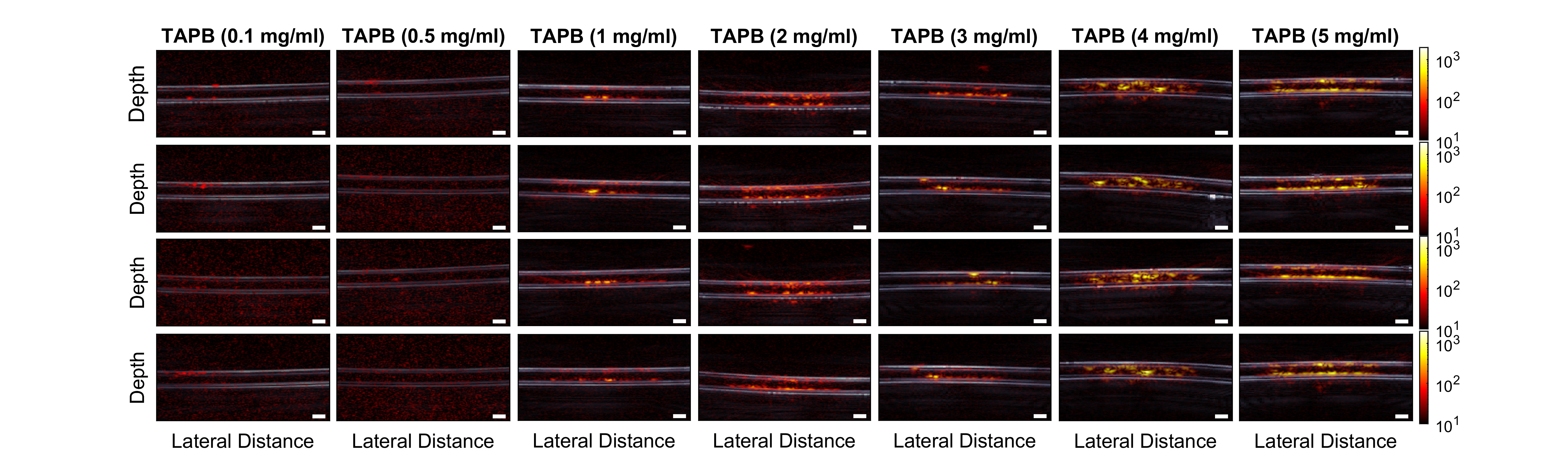


**Fig. S12.** Samples of fused imaged for TAPB cannulas for 0.1, 0.5, 1, 2, 3, 4, 5 mg/ml concentration.


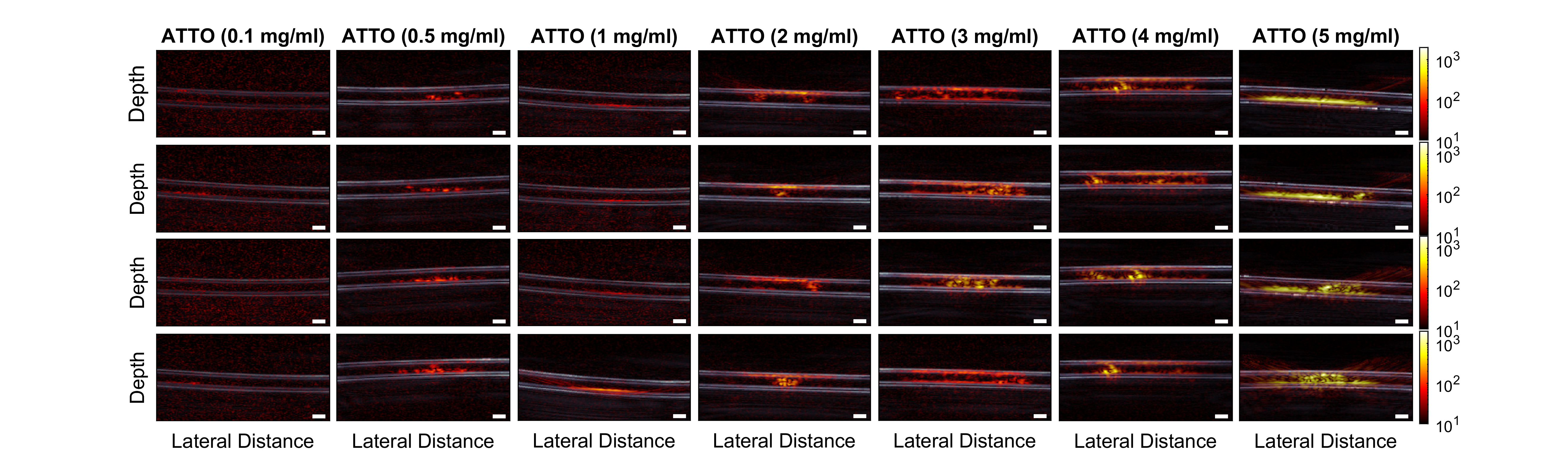


**Fig. S13.** Samples of fused imaged for ATTO cannulas for 0.1, 0.5, 1, 2, 3, 4, 5 mg/ml concentration.
